# Impact of In Ovo Leptin Injection and Dietary Protein Levels on Ovarian Growth Markers and Early Folliculogenesis in Post-Hatch Chicks (*Gallus gallus domesticus*)

**DOI:** 10.3390/biology13020069

**Published:** 2024-01-23

**Authors:** Sadequllah Ahmadi, Yuta Nemoto, Takeshi Ohkubo

**Affiliations:** 1 College of Agriculture, Ibaraki University, Ami 300-0393, Japan; sadequllah15@gmail.com (S.A.); 20a1066x@vc.ibaraki.ac.jp (Y.N.); 2 Faculty of Animal Science, Afghanistan National Agricultural Sciences and Technology University, Kandahar 3801, Afghanistan

**Keywords:** broiler, leptin, nutritional control, obesity, ovarian growth

## Abstract

**Simple Summary:**

Meat-type chickens are bred for rapid weight gain, which hinders reproduction and resembles a common hormonal disorder known as polycystic ovary syndrome (PCOS), causing infertility. We assessed how dietary protein and the hunger-controlling molecule leptin affect ovarian function in juvenile meat-type chicks. In ovo, leptin injection and diverse dietary protein levels had distinct effects on the molecules/genes that regulate ovarian growth and reproduction, potentially impacting follicle formation and egg production in adulthood. These findings could guide us on how dietary protein can affect animal reproduction, particularly in the poultry industry, regarding the biology of broiler chicks that are given high protein in their feed and its influence on reproduction and animal welfare.

**Abstract:**

Genetically bred for rapid growth, broiler breeder hens develop obesity and ovarian dysfunction when fed ad libitum, resembling a condition that resembles human polycystic ovary syndrome (PCOS). Nutritional control applies to post-hatched chicks from one week onward to prevent the development of a PCOS-like phenotype in adult broilers. This study investigated the impact of a growth marker, leptin, and post-hatch nutritional intake on early-life ovarian function. Fertile broiler eggs were injected in ovo with physiological saline solution or 5 µg of leptin and then incubated. After hatching, female chicks were fed ad libitum a diet containing low protein (17% low crude protein (LP)) or standard protein (22% standard crude protein (SP)). Tissues were collected from 7- and 28-day-old chicks for RT-qPCR and histological analysis. In contrast to the LP diet, the SP diet suppressed the mRNA expression of ovarian growth markers essential for folliculogenesis in post-hatched chicks. Leptin injection did not influence ovarian growth markers but increased pituitary gonadotropin transcripts in 7-day-old chicks fed with LP diet. No treatment effects on follicle activation were noted on day 7, but by day 28, in ovo leptin-treated LP-fed chicks exhibited a higher percentage of primary follicles. These changes may have resulted from the early upregulation of genes by leptin during the first week, including pituitary gonadotropins and ovarian leptin receptors. The decline in ovarian growth markers with the SP diet highlights the importance of precise post-hatch protein calculation, which may influence future ovarian function in animals. These findings may contribute to future dietary strategies to enhance broiler reproduction.

## 1. Introduction

Through intensive genetic selection, the progeny of commercial broiler breeders, known as the broiler breeder (BB) line, has been improved for rapid body weight gain of over 400% since the 1950s [1]. However, this strategy negatively affected the optimal reproduction of BB hens. As BB hens have a nearly insatiable appetite, ad libitum feeding of BB hens leads to increased lipid deposition, obesity, excessive ovarian growth, and severe ovarian dysfunction, including irregular follicular hierarchy, double ovulation, and decreased egg production [2,3]. Therefore, restricted feeding strategies have been employed for BB hens to optimize their reproductive efficacy [4,5]. However, energy deprivation causes hunger and stress in BB chickens, raising animal welfare concerns. The exact mechanism by which free access to feed intake diminishes reproductive performance in BB hens is poorly understood, but obesity triggered by ad libitum feeding might be responsible.

Obesity and higher abdominal fat deposition are closely associated with elevated plasma glucose, insulin, cholesterol, triacylglycerol, and phospholipid levels in BB hens [6,7]. Overfeeding alters the transcription of ovarian growth markers as well as plasma progesterone and estradiol levels, resulting in hyper-recruited prehierarchical follicles and irregular follicular hierarchy in adult birds [6,8,9,10]. This ovarian phenotype in BB hens resembles a human condition known as polycystic ovary syndrome (PCOS), which is associated with obesity, abnormal folliculogenesis and ovulation, and poor reproductive efficiency [7,11,12]. Patients with PCOS exhibit elevated plasma androgen and leptin levels and are primarily treated with dietary changes aimed at weight loss and menstrual cycle regulation. An identical strategy is used to normalize the follicular hierarchy and optimize reproduction in BB hens [5,11,13].

Adequate nutrition is essential to support optimal reproductive health from early life to reproductive age, with both the type and quantity of nutrition influencing reproduction. In neonatal mice, overfeeding results in obesity, the early onset of puberty, and premature ovarian aging [14]. Dietary protein restriction in the fetal and neonatal stages delays puberty and accelerates reproductive aging in female rat offspring [15]. Similarly, in BB hens fed ad libitum, ovarian expression of the steroidogenic markers cytochrome P450 aromatase (CYP19A1) and CYP11A was reported to be higher and was associated with increased follicular development at week 16 [16]. Another study revealed that nutrition modifies ovarian gene expression, with feed restriction significantly suppressing most steroid biosynthesis and neuropeptide-related genes in BB hens at 10 and 16 weeks of age [10].

Leptin regulates feed intake and energy expenditure in mammals. It also activates the hypothalamic–pituitary–gonadal (HPG) axis in response to stored body fat, and regulates reproduction [17,18,19,20]. Elevated plasma leptin levels due to obesity caused by neonatal overfeeding may be responsible for the early decline of ovarian reserves in adult rats [21], obese adolescents, and PCOS in adult females [22,23]. In chickens, leptin is primarily expressed in the HPG axis and digestive system [24,25] but is not detected in circulating blood, as it is in mammals [26]. Unlike mammalian leptin, bird leptin might not be directly responsible for satiety and energy expenditure through blood circulation, but it has a significant role in peripheral regulation of ovarian function [24,27,28]. In layer-type chickens, leptin has been shown to accelerate the onset of sexual maturation and egg production [29]. Leptin administration also advances primordial follicle growth in 7-day-old chicks by regulating the mRNA expression of key genes responsible for ovarian development, including pituitary luteinizing hormone (LH) and follicle-stimulating hormone (FSH), as well as ovarian follicle-stimulating hormone receptor (FSHR), CYP19A1, insulin-like growth factor 1 (IGF-1), IGF binding protein 2 (IGFBP2), anti-Müllerian hormone (AMH), and caspase 3 [27,28]. The association between gonadotropins/AMH and irregular follicle hierarchy is well documented in full-fed BB hens [4,30].

In chickens, follicle formation begins after the breakdown of germ cell cysts during the late embryonic stage. After hatching, individual oocytes are surrounded by a layer of epithelial granulosa cells, which form primordial follicles [31]. The mechanism of primordial follicle recruitment in chickens is not yet fully understood. However, studies in mammals have revealed that oocyte and primordial follicle growth is controlled by inhibitory or stimulatory intraovarian factors such as AMH, growth differentiation factor 9 (GDF9), bone morphogenetic proteins, activin, and the wingless-type MMTV integration site family (WNT) [32,33,34]. AMH interacts with IGF-I and FSHR and inhibits granulosa cell proliferation, whereas GDF9 and WNT ligands, including WNT5B, WNT6, and WNT11, stimulate it and are all highly expressed in the granulosa cells of small (<1 mm) follicles [33,34,35,36].

We hypothesized that in ovo leptin injection may mimic the conditions observed in obese pregnant hens, including elevated circulating leptin levels, possibly altering ovarian growth in chicks. We also assumed that post-hatch dietary proteins may influence ovarian growth markers during ovarian remodeling. Post-hatch days 7 and 28 are critical time points for ovarian development in chickens because the primary oocyte develops into a primordial follicle around post-hatch day 7, and its transition to a primary follicle occurs on post-hatch day 28 [31,37]. The present study examined the effects of in ovo administration of leptin and post-hatch dietary protein on the early regulation of ovarian growth markers and development in broiler chicks to explore the possibility of preventing a PCOS-like phenotype in adulthood.

## 2. Materials and Methods

### 2.1. Egg Incubation and In Ovo Injection

Commercial broiler (Ross 308) fertile eggs (*n* = 120) were obtained from a local hatchery (Matsumoto Chicken Farm, Ibaraki, Japan) and incubated at 37.5 °C and 60% relative humidity. In ovo injection was performed as previously described [29,38]. On the third day of incubation, the blunt end of the egg was sterilized with 70% ethanol and 10% povidone-iodine, the eggshell was drilled at the blunt end, and 200 μL of physiological saline solution (Otsuka Pharmaceutical Company, Tokyo, Japan) containing either 0 μg (*n* = 60) or 5 μg of recombinant mouse leptin (*n* = 60; Fujifilm-Wako, Osaka, Japan) was injected directly into the egg white. Subsequently, the eggs were sealed with cellophane tape and incubated. The type and dose of leptin were determined based on previous findings because authentic avian leptin was not available [28,39].

### 2.2. Animal-Rearing Conditions, Diet, and Sampling

After hatching, the chicks were sexed by feather discrimination [40], and the female chicks were divided into four battery cages. The chick house temperature was maintained at 30 °C for the first week and then decreased to 27 °C until the end of the experiment. In ovo saline or leptin-injected chicks were fed ad libitum with either commercial layer chick feed containing low protein (LP) (crude protein 17%, metabolizable energy 2850 kcal/kg) or commercial broiler starter feed containing standard protein (SP) (crude protein 22%, metabolizable energy 3000 kcal/kg) and water. The composition and ingredients of the experimental diets are listed in Supplementary Table S1. Four groups were generated based on the combination of in ovo injection and diet: Group 1 received the in ovo saline injection and LP diet; Group 2 received the in ovo saline injection and SP diet; Group 3 received the in ovo leptin injection and LP diet; and Group 4 received the in ovo leptin injection and SP diet. Each treatment had 12 replicates, except for the in ovo leptin injection with SP diet, which had 8 replicates. At 7 and 28 days of age, chicks were weighed and euthanized by decapitation of the atlanto-occipital joint (*n* = 6/group/day). The hypothalamus and pituitary were then collected in liquid nitrogen and stored at −80 °C until use. The ovary was also collected and cut equally into two fragments. One piece was snap-frozen in liquid nitrogen and stored at −80 °C for mRNA analysis, and the other was preserved in a 4% formaldehyde solution for histological analysis.

### 2.3. RNA Isolation, cDNA Synthesis, and Quantitative Real-Time PCR (qPCR)

Total RNA was isolated from the hypothalamus, pituitary, and ovaries on days 7 and 28 using ISOGEN II (Nippon Gene, Toyama, Japan) according to the manufacturer’s instructions. RNA concentrations were determined using a BioSpec-nano spectrophotometer (Shimadzu, Kyoto, Japan), and 400 ng of total RNA was reverse-transcribed into cDNA using ReverTra Ace qPCR RT Master Mix with genomic DNA remover (Toyobo, Osaka, Japan). The reverse transcription mixture was incubated at 37 °C for 15 min and then at 50 °C for 5 min, followed by reaction termination at 98 °C for 5 min. The synthesized cDNA was ultimately used for qPCR using the intercalator method to quantify the mRNA expression level of all genes except leptin in the collected tissues. qPCR was performed using GoTaq qPCR Master Mix (Promega, Madison, WI, USA) according to the manufacturer’s protocol in a 25 µL reaction. The analyzed genes in each tissue and primer sequences used for qPCR are listed in Table 1. PCR was performed in the CFX96 Real-Time System (Bio-Rad Laboratories, Hercules, CA, USA) as follows: initial denaturation at 95 °C for 10 s, followed by 40 cycles of 95 °C for 5 s and 60 °C for 30 s. Multiplex qPCR was employed to quantify leptin gene expression. A specific set of primers for leptin and chicken ribosomal protein S17 (cRPS17) with a probe was designed by Integrated DNA Technologies (Coralville, IA, USA). Multiplex qPCR was performed in a 20 μL reaction of 0.5 μM each forward and reverse primer with a 0.25 μM probe, 1 × PrimeTime Gene Expression Master Mix (Integrated DNA Technologies), and cDNA. PCR was performed in the CFX96 Real-Time System (Bio-Rad Laboratories) as follows: initial denaturation at 95 °C for 3 min, 40 cycles of 95 °C for 15 s, and 60 °C for 1 min. All qPCR samples were run in duplicate and analyzed using the 2^−∆∆Ct^ method [41].

### 2.4. Ovarian Histology

Fragmented ovaries preserved in 4% formaldehyde were embedded in paraffin wax. The paraffin-fixed ovaries were then sectioned serially at 5 μm thickness and stained with hematoxylin and eosin [42]. A light microscope (Olympus BX50, Tokyo, Japan) was used to count primordial follicles in the ovarian cortex of 7-day-old chicks and both the primordial and primary follicles in the ovarian cortex of 28-day-old chicks. Oocytes surrounded by a layer of granulosa cells (≤8 µm diameter) were counted as primordial follicles, and oocytes ringed by a layer of granulosa and theca cells (>0.8–1 mm diameter) were classified as primary follicles [31,43]. Follicles with a visible nucleus of the oocyte were counted in 8 sections in 3 microscopic fields of view (×20 magnification) for each of the 4–5 ovaries per group and averaged.

### 2.5. Statistical Analysis

The results were subjected to the Smirnov–Grubbs test to identify and eliminate outliers. Subsequently, a two-way ANOVA was employed for data analysis to check whether there was an interactive effect between feed and leptin administration, followed by post hoc Tukey’s honestly significant difference test. Statistical analyses were performed using the R package (www.r-project.org, accessed on 25 August 2023). The data were presented as mean ± standard error of the mean (SEM), with significance set at *p* < 0.05.

## 3. Results

### 3.1. Gene Expression Profile in the HPG Axis of 7-Day-Old Broilers Subjected to In Ovo Leptin Injection and Fed Different Protein Levels

**Figure 1:**
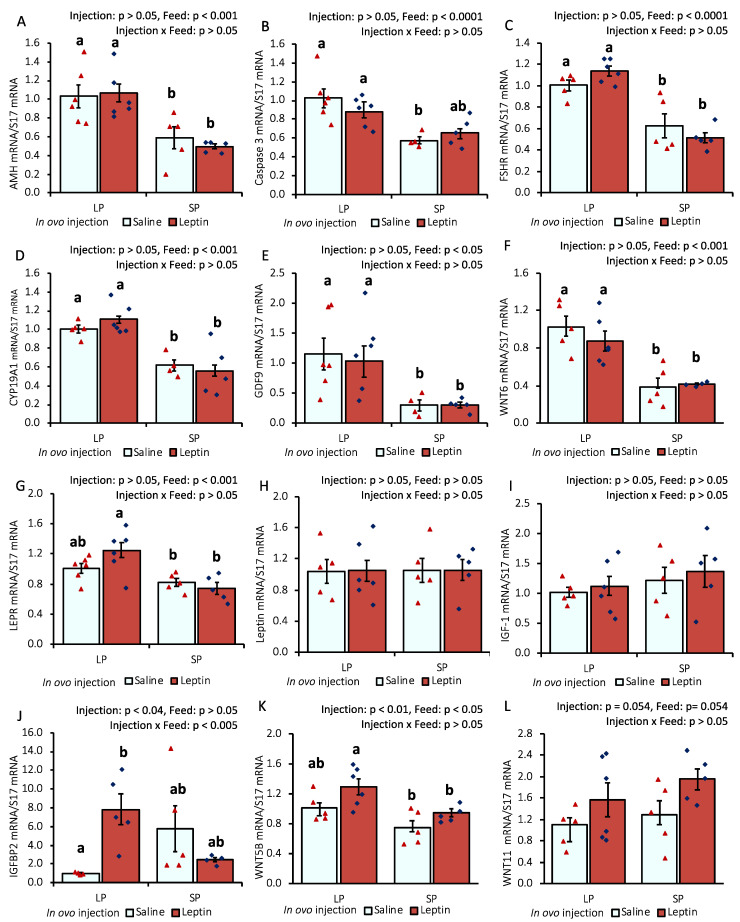
Ovarian mRNA expression of 7-day-old broiler chicks that received in ovo injection of leptin and different percentages of protein in the diet after hatching. (**A**) AMH, (**B**) caspase-3, (**C**) FSHR, (**D**) CYP19A1, (**E**) GDF9, (**F**) WNT6, (**G**) LEPR, (**H**) leptin, (**I**) IGF-1, (**J**) IGFBP2, (**K**) WNT5B, (**L**) WNT11. LP (low crude protein, 17%), SP (standard crude protein, 22%). Data represent the mean ± SEM; number of chicks per group as shown by individual data points. The outer layer was excluded when identified. Bars with different superscripts are significantly different (*p* < 0.05).

In response to different protein percentages in the feed, the mRNA expression of AMH, caspase 3, FSHR, CYP19A1, GDF-9, and WNT6 in 7-day-old chick ovaries showed differential regulation (*p* < 0.05), being downregulated with an SP-fed diet and upregulated with an LP-fed diet. In ovo leptin treatment did not significantly affect the mRNA regulation of these genes in the ovaries of 7-day-old chicks (Figure 1A–F). The gene expression levels of leptin receptor (LEPR) and IGFBP2 were significantly (*p* < 0.05) enhanced in the ovaries of leptin-injected LP-fed chicks compared with those in saline-treated LP-fed and SP-fed chicks. However, leptin mRNA transcript levels were unaffected by either leptin injection or diet. Ovarian IGF-1 gene expression remained unchanged in the treated groups, whereas the mRNA level of its binding factor 2, IGFBP2, showed a significant (*p* < 0.05) enhancement in the ovaries of leptin-treated LP-fed chicks (Figure 1G–J). Additionally, the mRNA expression of WNT5B was significantly increased by in ovo leptin injection in LP-fed chick ovaries compared with in SP-fed chick ovaries (Figure 1K). Furthermore, in ovo leptin injection tended to increase (*p* = 0.054) WNT11 mRNA expression in both LP-fed and SP-fed chick ovaries, compared to those injected with saline (Figure 1L).

**Figure 2:**
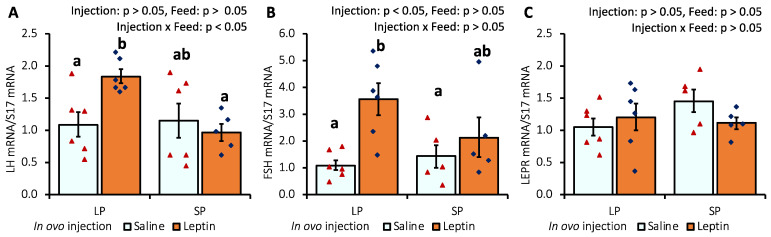
The mRNA expression of (**A**) LH, (**B**) FSH, and (**C**) LEPR in the pituitary of 7-day-old broiler chicks that received in ovo injection of leptin and different percentages of protein in the diet after hatching. LP (low crude protein, 17%), SP (standard crude protein, 22%). The data represent mean ± SEM; number of chicks per group as shown by individual data points. The outer layer was excluded when identified. Bars with different superscripts are significantly different (*p* < 0.05).

The gene expression levels of pituitary LH and FSH were significantly augmented only in LP-fed chicks that received in ovo leptin injection (Figure 2A,B). LEPR mRNA expression was not affected by leptin administration or diet (Figure 2C).

**Figure 3:**
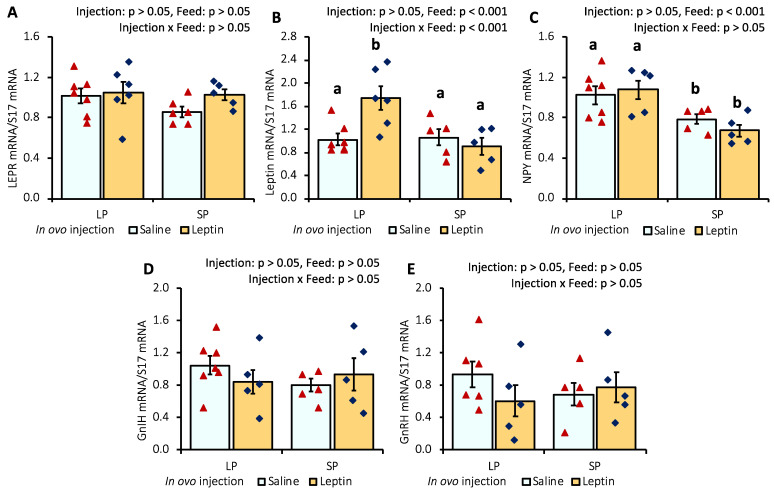
Hypothalamic mRNA expression of (**A**) LEPR, (**B**), leptin, (**C**) NPY, (**D**) GnIH, and (**E**) GnRH in 7-day-old broiler chicks that received in ovo injection of leptin and different percentages of protein in the diet after hatching. LP (low crude protein, 17%), SP (standard crude protein, 22%). Data represent the mean ± SEM; number of chicks per group as shown by individual data points. The outer layer was excluded when identified. Bars with different superscripts are significantly different (*p* < 0.05).

In the hypothalamus of in ovo-injected 7-day-old chicks, leptin injection did not modify LEPR gene expression, whereas leptin expression was significantly enhanced in the leptin-injected LP group (Figure 3A,B). In contrast to the LP diet, the SP diet significantly (*p* < 0.05) downregulated neuropeptide Y (NPY) mRNA expression in the hypothalamus (Figure 3C). Moreover, the mRNA expression of gonadotropin-inhibitory hormone (GnIH) and gonadotrophin-releasing hormone (GnRH) was not affected by either leptin injection or different protein diets (Figure 3D,E).

### 3.2. Ovarian Histology of 7-Day-Old Broilers

**Figure 4:**
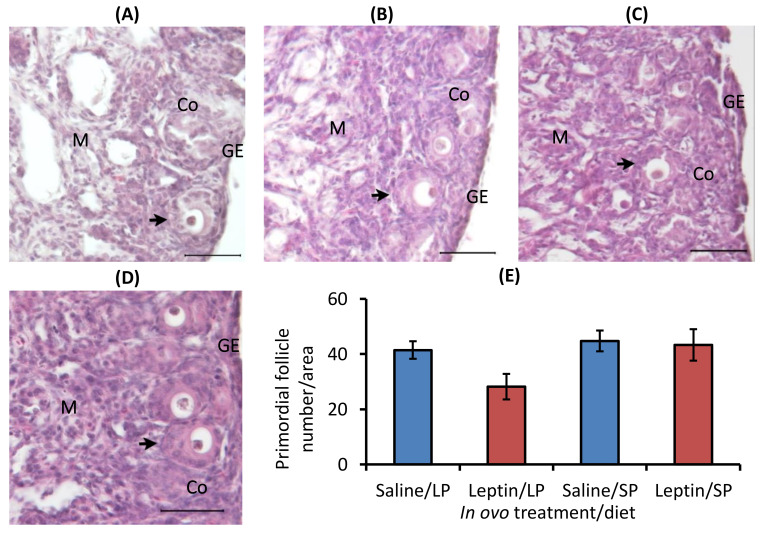
Representative ovary sections and follicle numbers of 7-day-old broilers that received in ovo injection of leptin and different percentages of protein in the diet after hatching. (**A**) LP (low crude protein, 17%) diet with in ovo saline injection, (**B**) LP diet with in ovo leptin injection, (**C**) SP (standard crude protein, 22%) diet with in ovo saline injection, and (**D**) SP diet with in ovo leptin injection. (**E**) Average number of primordial follicles in the ovary. GE—germinal epithelium; CO—cortex; M—medulla. Solid arrows indicate primordial follicles. Scale bars denote 50 µm. Data represent the mean ± SEM of five individual chick ovaries in each treatment group.

In ovo injection of leptin or different levels of protein in the diet did not significantly alter the primordial follicle number in 7-day-old broiler chicks. The ovarian histology and primordial follicle number of 7-day-old broiler chicks are shown in Figure 4.

### 3.3. Gene Expression Profile in the HPG Axis of 28-Day-Old Broilers

**Figure 5:**
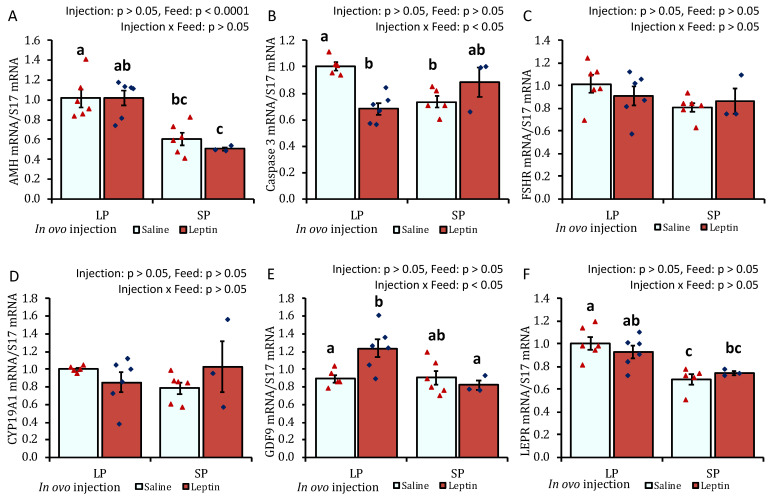
The ovarian mRNA expression of 28-day-old broiler chicks that received in ovo injection of leptin and different percentages of protein in the diet after hatching. (**A**) AMH, (**B**) caspase-3, (**C**) FSHR, (**D**) CYP19A1, (**E**) GDF9, and (**F**) LEPR. LP (low crude protein, 17%), SP (standard crude protein, 22%). Data represent the mean ± SEM; number of chicks per group as shown by individual data points. The outer layer was excluded when identified. Bars with different superscripts are significantly different (*p* < 0.05).

**Figure 6:**
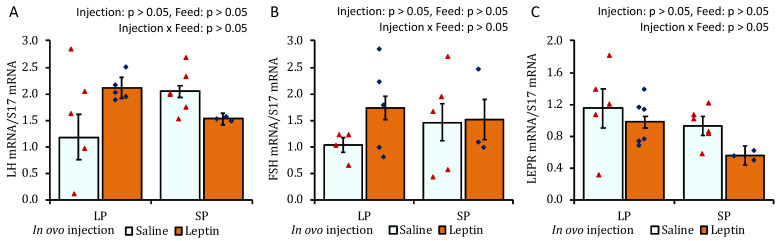
The mRNA expression of (**A**) LH, (**B**) FSH, and (**C**) LEPR in the pituitary of 28-day-old broiler chicks that received in ovo injection of leptin and different percentages of protein in the diet after hatching. LP (low crude protein, 17%), SP (standard crude protein, 22%). Data represent the mean ± SEM; number of chicks per group as shown by individual data points. The outer layer was excluded when identified.

The ovaries of SP-fed 4-week-old chicks showed lower AMH mRNA expression than those of LP-fed chicks (Figure 5A). In contrast to the LP-fed saline-treated group, a significant decline in mRNA expression was observed for leptin in the LP-fed leptin-administrated and SP-fed saline-treated groups (Figure 5B). No specific effects of either the protein diet or leptin injection were observed on ovarian FSHR and CYP19A1 gene expression on day 28 (Figure 5C,D). There was a significant increase in the mRNA expression of GDF9 in the ovaries of leptin-treated LP-fed chicks (Figure 5E). The ovaries of SP-fed chicks exhibited lower LEPR mRNA expression than those of LP-fed chicks that received saline injection (Figure 5F). In ovo leptin administration and post-hatch variations in dietary protein levels did not affect pituitary LH, FSH, or LEPR mRNA expression in 4-week-old broilers (Figure 6A–C).

### 3.4. Ovarian Histology of 28-Day-Old Broilers

**Figure 7:**
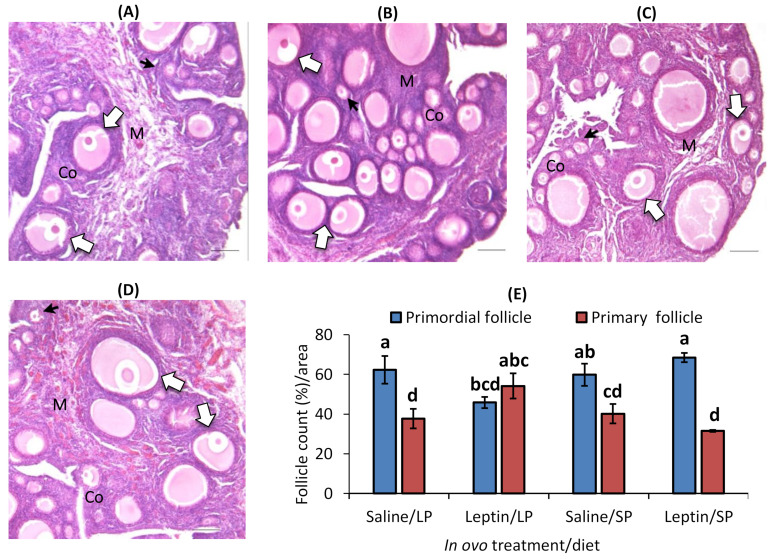
Representative ovary sections and follicle numbers of 28-day-old broilers that received in ovo injection of leptin and different percentages of protein in the diet after hatching. (**A**) LP (low crude protein, 17%) diet with in ovo saline injection, (**B**) LP diet with in ovo leptin injection, (**C**) SP (standard crude protein, 22%) diet with in ovo saline injection, and (**D**) SP diet with in ovo leptin injection. (**E**) Average percentage of primary and primordial follicles in the ovary. GE—germinal epithelium; CO—cortex; M—medulla. Solid and open arrows indicate primordial and primary follicles, respectively. Scale bars denote 50 µm. The data represent the mean ± SEM of five individual chick ovaries in each treatment group. Bars with different superscripts show significant differences (*p* < 0.05).

In contrast to the primary follicle, the primordial follicle percentage was higher in each group except the leptin-administered LP-fed group. Interestingly, the percentage of primordial follicles was significantly lower and that of primary follicles was higher in the leptin-administered LP-fed group than in the other groups (Figure 7A–E).

## 4. Discussion

Ovarian development and folliculogenesis are continuous processes that extend from the embryonic stage to the reproductive lifespan, and energy availability significantly affects this development. However, information on the effects of energy intake on ovarian function in early life (1–4 weeks of age) in animal models exhibiting PCOS during adulthood is unavailable. We found that the level of dietary protein alters the mRNA transcripts of HPG-axis genes, particularly in the ovaries of early life broiler chicks, potentially affecting future folliculogenesis. Our results are consistent with those of other studies and suggest that maternal or neonatal imbalanced nutritional intake influences folliculogenesis and reproduction during the pubertal and prepubertal stages in animals, including BB hens [10,44,45].

In this study, different levels of protein intake significantly influenced AMH gene expression in the ovaries of 7- and 28-day-old chicks (Figure 1A and Figure 5A). Decreased AMH mRNA from higher protein intake may enhance granulosa cell proliferation, which is essential for primordial follicle activation, by altering follicular sensitivity to FSH in mouse ovaries [46,47]. Although the suppression of AMH in SP-fed 7-day-old chicks did not enhance the primordial follicle number (Figure 4), the early age alteration of AMH by protein intake might influence future follicle reserves. This is supported by a study in overfed neonatal mice in which it was observed that the suppression of AMH by higher energy intake was associated with an early decline in follicles analyzed at puberty [21]. In line with our observations (Figure 4 and Figure 7), the same author reported that neonatal overfeeding did not affect follicle number as early as postnatal day 14, but decreased it significantly in adulthood [21]. In addition, a previous study reported that AMH and GDF9 gene expression levels were not altered by restricted or full feeding in 16-week-old BB hens [16] and adult rats [21], further suggesting that these markers are more sensitive to nutritional intake at a young age compared with prepubertal age and may affect primordial follicle pool assembly at a young age. AMH was observed to hinder the assembly of primordial follicles, resulting in a reduction in the initial pool size in a rat ovarian organ culture, and to induce modifications in the ovarian transcriptome during primordial follicle assembly, influencing the expression of more than 200 genes [48]. Moreover, in response to lower or higher protein diets, the AHM mRNA profile was positively correlated with the apoptotic marker, caspase 3, in the ovaries of 7-day-old chicks (Figure 1A,B and Figure 5A,B). A similar correlation between AMH and caspase-3 has also been reported previously in cultured granulosa cells [49].

Granulosa cell growth significantly enhances the recruitment of primordial follicles [34], for which the expression of intraovarian growth factors such as FSHR, CYP19A1, GDF9, WNTs, and IGF-1 is essential in the ovary [33,35,50,51]. The mRNA profile of this cohort of genes was not induced by protein intake or leptin injection in the ovaries of our experimental birds; instead, FSHR, CYP19A1, GDF9, and WNT6 exhibited a similarly low mRNA expression trend as that observed for AMH and caspase 3 genes in the ovaries of 7-day-old broilers (Figure 1C–I). To the best of our knowledge, little is known about this intricate regulation of nutrient intake at a young age in vertebrates, including chickens. However, our observations suggest that other central or intraovarian growth factors may play a role in maintaining the dormancy of primordial follicles at this age. This could involve suppression of the mRNA expression of ovarian FSHR, CYP19A1, GDF9, and WNT11 as a negative feedback response to decreased AMH and caspase-3 in response to higher protein intake and excessive growth (Figure 1 and Supplementary Figure S1). In addition, with the exception of the AMH and LPER genes, such differential effects of dietary protein on the expression levels of caspase 3, FSHR, CYP19A1, and GDF9 were not detected on day 28 (Figure 5). This suggests that ovarian growth markers are sensitive to dietary protein levels at an early age. Interestingly, a similar expression trend in hypothalamic NPY with ovarian AHM, FSHR, CYP19A1, GDF9, and WNT11 (Figure 1 and Figure 2) was observed, whose expressions were increased in LP-fed chick ovaries and decreased in SP-fed chick ovaries on day 7. The direct impact of NPY on the regulation of these ovarian markers is not well defined in animals. However, NPY plays a critical role in food intake and reproductive behavior in mammals [52] and chickens [53]. The change in NPY gene expression by different protein diets was consistent with the findings of other studies [54,55]; however, differentially expressed NPY did not modify GnRH mRNA expression (Figure 3), which was observed in adult mice in a previous study [56]. The interaction between NPY and GnRH has not been explored in juvenile chicks. Furthermore, the modification of WNT ligand mRNA in the ovaries of 7-day-old chicks, induced by either leptin injection or diverse protein intake (Figure 1F,K), indicated the interaction of this pathway with intraovarian growth factors. This interaction may have implications for the proliferation of granulosa cells in the ovary but has not been studied in chickens. However, research in mammals has indicated that canonical WNT signaling plays a permissive role in facilitating the transition of pregranulosa cells to granulosa cells and is essential for supporting oocyte growth [33,57].

The in ovo injection of leptin altered the ovarian LEPR and IGFBP2 mRNA levels but not the levels of AMH, FSHR, Cyp19A1, leptin, and IGF-1 mRNA in 7-day-old broiler chicks (Figure 1). Our previous study demonstrated that intraperitoneal leptin administration directly affected the mRNA expression of these genes in layer chicks [27,28]. This discrepancy may be due to differences in the types of chickens. Leptin injection did not alter LEPR mRNA expression in the hypothalamus or pituitary of the treated groups on day 7. However, in LP-fed chicks, hypothalamic leptin and ovarian LEPR expression were increased after leptin injection, demonstrating a positive correlation with elevated LH and FSH mRNA expression in the pituitary and ovary of LP-fed, leptin-administered chicks on day 7 (Figure 2A,B and Figure 3B) but not on day 28 (Figure 6A–C), as observed previously in layer chicks [27]. Such regulation of genes by leptin might initiate primordial follicle transition into primary follicles because the rise in gonadotropin activity dramatically induces the reproductive axis within the first week of hatching [58]. LH treatment in chick embryos has been shown to accelerate oogonia proliferation into oocytes and increase the number of follicles by one week of age [59], and leptin administration has been shown to enhance primordial follicle growth [28] and advanced sexual maturation in birds [29,38]. Considering these findings, the increase in gonadotropins by leptin on day 7 (Figure 3C,D) and ovarian GDF9 on day 28 (Figure 5E) in LP-fed chicks may have resulted in a higher percentage of primary follicles, as observed on day 28 (Figure 7). However, a similar effect of leptin injection was not found in the SP-fed chick pituitary or in histological changes in their ovaries, suggesting that increased dietary protein, leading to higher body weight (Supplementary Figure S1), may affect the physiological action of leptin and prompt earlier ovarian growth. In addition, excessive follicle activation may not be advantageous at this age in broilers, as the follicles will not enter cyclic development and will become atretic, thereby decreasing the follicle reserves needed at the pubertal stage [60]. Although we did not analyze the effects of prolonged protein in this study, we propose that early age dietary protein regulation of genes may impact future folliculogenesis in broilers and requires further research, especially for broiler and layer breeder lines. In addition, blocking leptin during the embryonic or post-hatch stages could help explore its interaction with ad libitum feeding on follicle growth and hen fertility. The differences between our results and the results of other studies may have arisen from variations in the injected leptin type, the timing of injection, nutritional responses, and genetic characteristics of the examined birds.

## 5. Conclusions

The results of the present study showed that dietary protein intake after hatching in broilers alters the mRNA expression of ovarian growth markers that are crucial for follicle development, potentially affecting future folliculogenesis. In ovo, leptin administration enhanced pituitary and ovarian transcripts and advanced follicle transition in 4-week-old broilers fed a low (LP) but not standard (SP) protein diet. This suggests that the dietary protein proportion may impact HPG-axis genes related to ovarian growth from a young age, which might pose challenges during the reproductive age in animals. These findings may guide future dietary strategies to enhance reproduction in PCOS-phenotype BB hens. However, additional studies are necessary to investigate the prolonged effects of protein intake on ovarian development in this breed.

## Figures and Tables

**Table 1:** Primer sequences used for quantitative real-time PCR.

Target	Gene	Forward Sequence (5′ to 3′)	Reverse Sequence (5′ to 3′)	Amplicon Size, bp	Accession Number
All tissues	Leptin	GCAGCAAACTGCAAAGGTTATC	ACGATTGAGGCGATTCCAAC	100	KT970642.1
		Probe: 56-FAM/CCTCTACTG/ZEN/CTGCAGCTGCGAA/31ABkFQ			
	cRPS17	CTTCATCAGGTGGGTGACATAC	AACGACTTCCACACCAACAA	102	NM204217.2
		Probe: 5HEX/CAGCAAGAA/ZEN/GCTGCGCAACAAGAT/31ABkFQ			
	LEPR	TCTGCTCAGAGGTGTGGGAT	CTGAAACTGCGGCACGTATG	103	NM204323
Hypothalamus	NPY	GGCACTACATCAACCTCATC	CTGTGCTTTCCCTCAACAA	93	NM_205473.2
	GnRH	ACACTGGTCTTATGGCCTGCA	ATTCAGCCTTCTGCCCTTCTC	116	NM001080877.1
	GnIH	GCATGGTATGTGCCTAGATGAACTAAT	TCCTCTGCTTTTCCTCCAAGATA	110	NM204363.1
Pituitary	LH	AACGTAACGGTGGCGGTG	AGGCCGTGGTGGTCACAG	64	HQ872606.1
	FSH	CCACGTGGTGCTCAGGATACT	AGGTACATATTTGCTGAACAGATGAGA	84	NM204257.1
Ovary	CYP19A1	CCAGTTGCCACAGTGCCTAT	CCTGGCCCTGGTATTGATGA	89	NM001001761.2
	FSHR	ACCTGCCTGGATGAGCTAAAT	ATCCAAAACAACAGGCCCGA	96	NM205079.1
	AMH	CCCCTCTGTCCCTCATGGA	CGTCATCCTGGTGAAACACTTC	71	NM205030.2
	GDF9	GAGACTTTCACTCGGTGGATT	ATGCTGGGACATACTTGGC	178	NM_206988.3
	Caspase 3	GGAAGCATCTCTACTTGGGGG	CTCCCCCTTTCTGAGGACCA	70	NM204725.1
	IGF-1	CTTCAGTTCGTATGTGGAGACA	GATTTAGGTGGCTTTATTGGAG	167	NM001004384.3
	IGFBP2	CTGGTGCAGGGACAGGG	ACGTGGTTCTCAGCAAGGAT	122	NM2053
	WNT5B	CGGGGATAACGTGGAGTACG	GTTCATCAGCATGCGAGCCT	112	NM_001037269.1
	WNT6	CGACGTGCAGTTTGGCTATG	CGTGGCATTTGCACTCTGTC	155	NM_001007594.3
	WNT11	AGAGGGGATCTGGACTCAGC	CCGAGGGGAAAATAGGAGGC	118	XM_046906641.1
	S17	GACCCGGACACCAAGGAAAT	GCGGCGTTTTGAAGTTCATC	100	NM204217.1

## Data Availability

None of the data were deposited in an official repository.

## Supplementary Materials

The following supporting information can be downloaded at: https://www.mdpi.com/article/10.3390/biology13020069/s1, Figure S1: Body weight; Table S1: Diet composition.
