# Supplementary material for: Impact of In Ovo Leptin Injection and Dietary Protein Levels on Ovarian Growth Markers and Early Folliculogenesis in Post-Hatch Chicks (Gallus gallus domesticus)
Source: Biology (Basel). 2024 Jan 23;13(2):69. doi: 10.3390/biology13020069 (PMC10886161; doi:10.3390/biology13020069)
Supplement: Supplementary file 1 [file biology-13-00069-s001.zip › Supplementary Figure S1.pdf]

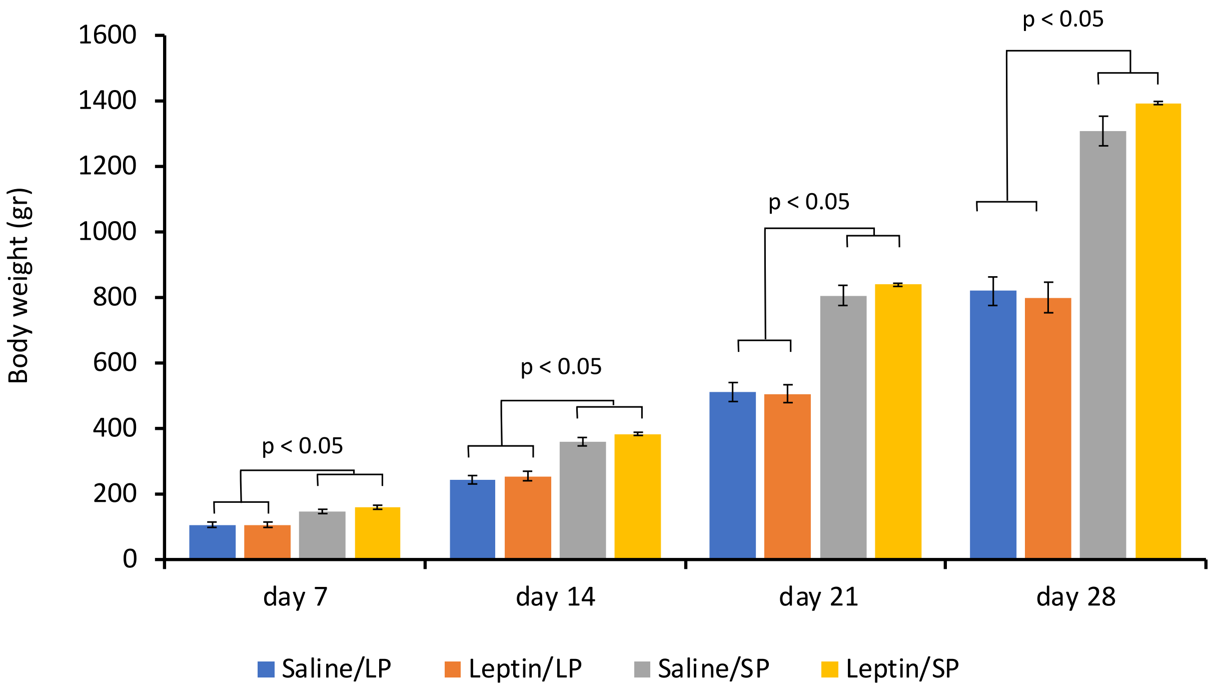

Supplementary Figure S1. Weekly body weight of broiler chicks after receiving *in ovo* leptin injection and diverse protein levels in the diet after hatch, LP (low crude protein, 17%) and SP (standard crude protein, 22%). The data represent the mean ± SEM, with n = 12 on day 7 and n = 6 after day 7.
