# Supplementary material for: Impact of In Ovo Leptin Injection and Dietary Protein Levels on Ovarian Growth Markers and Early Folliculogenesis in Post-Hatch Chicks (Gallus gallus domesticus)
Source: Biology (Basel). 2024 Jan 23;13(2):69. doi: 10.3390/biology13020069 (PMC10886161; doi:10.3390/biology13020069)
Supplement: Supplementary file 1 [file biology-13-00069-s001.zip › Supplementary Table S1.pdf]

Supplementary Table S1. Type and composition of the experimental diets from day 1 to 28.

**Ingredients (%)**	**Broiler feed** (standard crude protein (SP))	**Layer feed** (low crude protein (LP))
Crude protein	22.0	1 7.0
Crude fat	3.0	2.5
Crude fiber	5.0	6.0
ash	8.0	9.0
Calcium	0.80	0.80
Phosphorus	0.55	0.50
Metabolic energy/kg	3,000 Kcal	2,850 Kcal
